# Characterization of *Mycoplasma gallisepticum* pyruvate dehydrogenase alpha and beta subunits and their roles in cytoadherence

**DOI:** 10.1371/journal.pone.0208745

**Published:** 2018-12-10

**Authors:** Jingjing Qi, Fanqing Zhang, Yu Wang, Ting Liu, Lei Tan, Shaohui Wang, Mingxing Tian, Tao Li, Xiaolan Wang, Chan Ding, Shengqing Yu

**Affiliations:** 1 Shanghai Veterinary Research Institute, the Chinese Academy of Agricultural Sciences (CAAS), Shanghai, PR China; 2 College of Veterinary Medicine, Yangzhou University, Yangzhou, Jiangsu, PR China; 3 Jiangsu Co-innovation Center for Prevention and Control of Important Animal Infectious Diseases and Zoonoses, Yangzhou, Jiangsu, PR China; Miami University, UNITED STATES

## Abstract

*Mycoplasma gallisepticum* is a causative agent of chronic respiratory disease in chickens, typically causing great economic losses. Cytoadherence is the critical stage for mycoplasma infection, and the associated proteins are important for mycoplasma pathogenesis. Many glycolytic enzymes are localized on the cell surface and can bind the extracellular matrix of host cells. In this study, the *M*. *gallisepticum* pyruvate dehydrogenase E1 alpha subunit (PDHA) and beta subunit (PDHB) were expressed in *Escherichia coli*, and their enzymatic activities were identified based on 2,6-dichlorophenol indophenol reduction. When recombinant PDHA (rPDHA) and recombinant PDHB (rPDHB) were mixed at a 1:1 molar ratio, they exhibited strong enzymatic activity. Alone, rPDHA and rPDHB exhibited no or weak enzymatic activity. Further experiments indicated that both PDHA and PDHB were surface-exposed immunogenic proteins of *M*. *gallisepticum*. Bactericidal assays showed that the mouse anti-rPDHA and anti-rPDHB sera killed 48.0% and 75.1% of mycoplasmas respectively. A combination of rPDHA and rPDHB antisera had a mean bactericidal rate of 65.2%, indicating that rPDHA and rPDHB were protective antigens, and combining the two sera did not interfere with bactericidal activity. Indirect immunofluorescence and surface display assays showed that both PDHA and PDHB adhered to DF-1 chicken embryo fibroblast cells and adherence was significantly inhibited by antisera against PDHA and PDHB. Adherence inhibition of *M*. *gallisepticum* to DF-1 chicken embryo fibroblast cells was 30.2% for mouse anti-rPDHA serum, 45.1% for mouse anti-rPDHB serum and 72.5% for a combination of rPDHA and rPDHB antisera, suggesting that rPDHA and rPDHB antisera may have synergistically interfered with *M*. *gallisepticum* cytoadherence. Plasminogen (Plg)-binding assays further demonstrated that both PDHA and PDHB were Plg-binding proteins, which may have contributed to bacterial colonization. Our results clarified the enzymatic activity of *M*. *gallisepticum* PDHA and PDHB and demonstrated these compounds as Plg-binding proteins involved in cytoadherence.

## Introduction

*Mycoplasma gallisepticum* is one of the most important avian pathogens. It is the primary agent of chronic respiratory disease in chickens and infectious sinusitis in turkeys, causing great economic losses in the poultry industry worldwide [1]. Previous studies showed that *M*. *gallisepticum* invades DF-1 chicken embryo fibroblast cells (named DF-1 cells thereafter) in *vitro* [2], passes through the respiratory mucosal barrier, enters the bloodstream and spreads throughout the body [3]. Cytoadherence is the initial stage for mycoplasmas to colonize and infect host cells [4] and is essential for mycoplasma virulence [3]. Investigation of proteins involved in cytoadherence is important for a better understanding of mycoplasma pathogenesis. Several cytoadherence-related proteins of *M*. *gallisepticum* have been reported, such as GapA and CrmA [5–7], MGC2 [8], PvpA [9], and the OsmC-like protein MG1142 [10]. In addition, studies revealed that some glycolytic enzymes, including glyceraldehyde-3-phosphate dehydrogenase (GAPDH) [11–14] and α-enolase (Eno) [15–18], are on the surface of mycoplasmas. They are involved in cytoadherence through interactions with host components such as plasminogen (Plg) [17–20], fibronectin (Fn) [11, 18], mucin [12] and β-actin [14, 16]. In *M*. *gallisepticum*, some metabolic enzymes are also cytoadhesins including triosephosphate isomerase [21], Eno [22], and pyruvate kinase [23]. These multifunctional glycolytic enzymes are called moonlighting proteins [24]; whether other moonlighting proteins exist in *M*. *gallisepticum* is not clear. The multiple functions of proteins may be a way to compensate for limited genetic resources. Based on this hypothesis, further investigation of moonlighting proteins is helpful for enrichment of the adhesion-related proteins in *M*. *gallisepticum* and for better understanding the organization of the parasitic lifestyle of mycoplasmas.

In bacteria, the pyruvate dehydrogenase complex (PDHc) is an important glycolytic enzyme complex that converts pyruvic acid to acetyl-CoA and catalyses the reduction of NAD+ to NADH. In addition to enzyme activity, the pyruvate dehydrogenase E1 subunit (PDH E1) is an immunogenic protein from many mycoplasma species such as *M*. *agalactiae* [25], *M*. *bovis* [26], *M*. *pneumoniae* [27], *M*. *hyopneumoniae* [28], *M*. *mycoides* subsp. *mycoides* SC [29] and *M*. *mycoides* subsp. *capri*. [30, 31]. The PDH E1 alpha subunit (PDHA) of *M*. *pneumoniae* is a membrane-associated protein [32] and was confirmed as an P1 adhesin-complexed protein [33]. The PDH E1 beta subunit (PDHB) of *M*. *pneumoniae* is a cell surface-located protein that binds human Fn and plg [27,34]. Both PDHA and PDHB of *M*. *pneumoniae* bind to human Plg [35, 36] and other human extracellular matrix (ECM) proteins [37]. In addition, PDHA and PDHB of *M*. *pneumoniae* bind to human lung epithelial cells and binding is reduced significantly by anti-plasminogen. This result indicates that PDH E1 might be involved in colonization of the respiratory tract [36]. However, PDH E1-mediated cell adherence is rarely reported in other mycoplasmas. Here, we investigated the enzyme activities, subcellular localization, immunogenicity, cytoadherence and Plg-binding ability of *M*. *gallisepticum* PDHA and PDHB. The results may provide a molecular basis for further study of their function in *M*. *gallisepticum* pathogenesis.

## Materials and methods

### Bacterial strains, vectors, sera, cell lines, and cell culture

*M*. *gallisepticum* strain R_*low*_ (CVCC 1651) was obtained from the China Veterinary Culture Collection Center (CVCC, Beijing, China) and cultured in mycoplasma broth base (Haibo, Qingdao, China) with 10% horse serum (Thermo Fisher Scientific, Waltham, MA, USA) at 37°C in a 5% CO_2_ atmosphere. The His-tag vector pET-28a (+) (Novagen, Madison, WI, USA) was used for DNA manipulation. The vector pET28a-InaZN-EGFP was used for the surface display system and named as pIGN. The pIGN vector contains nucleotide sequences for the N-terminal domain of ice nucleation (InaZN) and enhanced green fluorescent protein (EGFP) and was previously constructed in our lab [38]. The *M*. *gallisepticum*-infected and *M*. *gallisepticum*-negative chicken sera were from CVCC. *Escherichia coli* strains DH5α and BL21 (DE3) (Tiangen, Beijing, China) were used as host strains for gene cloning and recombinant protein expression; after transformation with recombinant expression vectors, *E*. *coli* were grown in Luria-Bertani (LB) broth or on LB agar plates supplemented with 50 μg mL^−1^ kanamycin at 37°C. DF-1 cells were from American Type Culture Collection (ATCC, Manassas, VA, USA) and cultured in high-glucose Dulbecco’s modified Eagle’s medium (DMEM; GE Healthcare Life Science, HyClone, Logan, UT, USA) containing 10% foetal bovine serum (FBS; Gibco, Carlsbad, CA, USA), 100 IU mL^−1^ penicillin, and 100 μg mL^−1^ streptomycin, at 37°C in a 5% CO_2_ atmosphere. DF-1 cells used in this study were confirmed to be mycoplasma-free using PCR PromoKine Mycoplasma Test kits I/C (PromoCell, Heidelberg, Germany) according to the manufacturer’s protocol.

### Cloning and expression of *M*. *gallisepticum pdhA* and *pdhB* genes

*M*. *gallisepticum* strain R_*low*_ cultures at logarithmic growth were harvested by centrifugation at 12,000 ×*g* for 10 min, and genomic DNA was extracted using TIANamp bacterial genomic DNA kits (centrifugal column type; Tiangen, Beijing, China). Using the *M*. *gallisepticum* strain R_*low*_ genome sequence (NC_004829.2), gene sequences for *pdhA* (MGA_RS02765) and *pdhB* (MGA_RS02760) were extracted and analysed. Five TGA sites were found in the *pdhA* gene, and two in the *pdhB* gene; this sequence encodes tryptophan in mycoplasmas but is a stop codon in *E*. *coli*. To change TGA to TGG, overlapping PCR was conducted for site-directed mutagenesis using primers in Table 1 (A1F to A6R for *pdhA*; B1F to B3R for *pdhB*). Full-length *pdhA* and *pdhB* gene fragments were subcloned into pET-28a (+) at the *Bam*H I/*Xho* I sites for *pdhA* and *Bam*H I/*Eco*R I sites for *pdhB*. Recombinant PDHA (rPDHA) and PDHB (rPDHB) proteins were expressed in *E*. *coli* BL21 (DE3) by incubation with 0.5 mM isopropyl β-D-1-thiogalactopyranoside (IPTG) at 37°C for 6 h, purified using Ni-NTA His-Bind Resin kits (Novagen) and analysed by sodium dodecyl sulfate-polyacrylamide gel electrophoresis (SDS-PAGE). The protein concentrations of purified rPDHA and rPDHB were determined using BCA protein quantification kits (Thermo Fisher Scientific-Pierce, Rockford, IL, USA).

**Table 1:** Primers for overlapping PCR of *pdhA* and *pdhB*.

Primers	Sequences (5'→3')
A1F	C*GGATCC*^a^ATGGCAATTATTGTTAAAAAC
A1R	CGTTG**C**^b^CAAGTTAACATCTTCTTGTCAAG
A2F	GTTAACTTG**G**^b^CAACGTTCAGGTAAGATG
A2R	GGTACTAA**C**^b^CAGTCTTTCTTAGTCATTGC
A3F	GACTG**G**^b^TTAGTACCAGCTTTCAGATCAGG
A3R	CATTACCGTT**C**^b^CAGTAAAGCATTAATTGG
A4F	GCTTTACTG**G**^b^AACGGTAATGAAAAAGGTAAC
A4R	GACAGTTTG**C**^b^CACTTGTGAATTGAAGC
A5F	CACAAGTG**G**^b^CAAACTGTCTTCTGTGTAAAC
A5R	GCTTCGT**C**^b^CATAGACCTTTAGCTGTTA
A6F	GGTCTATG**G**^b^ACGAAGCTAAAGAAAAAAC
A6R	CG*CTCGAG*^a^TTATTTGTCTCCAAAG
B1F	C*GGATCC*^a^ATGAGTGATAAAATTATCG
B1R	CGGAGTATC**C**^b^CATACTCGATCAGCAC
B2F	CGAGTATG**G**^b^GATACTCCGATCTCAGAAG
B2R	CTGTGTT**C**^b^CAGTCAATTGGTGAGATTG
B3F	CACCAATTGACTG**G**^b^AACACAGTTTTAGG
B3R	CG*GAATTC*^a^CTAAGCTAGTAATTCGTTAATTG

^a^ Sites of *Bam*H I (GGATCC), *Xho* I (CTCGAG) and *Eco*R I (GAATTC) are in italics.

^b^ Nucleotide mutation sites are in bold.

### Detection of enzyme activity

PDHc E1 subunits catalyse the thiamine pyrophosphate (TPP)-dependent decarboxylation of pyruvate, producing 2α-hydroxyethylidene-TPP, which reduces 2,6-dichlorophenol indophenol (2,6-DCPIP, Sigma-Aldrich, St. Louis, MO, USA) from blue to colourless; this change can be monitored by a spectrophotometer [39]. PDHA and PDHB are two components of the PDHc E1 subunit. The enzymatic activity of rPDHA and rPDHB was determined by measuring decreases in optical density (OD) at 600 nm (OD_600_), reflecting the reduction of 2,6-DCPIP, according to Nemeria *et al*. [39], with a few modifications. Reaction buffer contained 0.1 mM 2,6- DCPIP (Sigma), 0.2 mM TPP (Sigma), 2.0 mM sodium pyruvate, and 0.1 mM MgCl_2_ and 50 mM KH_2_PO_4_ (pH 7.0), pre-warmed at 30°C before 1 mL reaction buffer was added with 10 μg rPDHA, 10 μg rPDHB, or a mixture of rPDHA (4.6 μg) and rPDHB (5.4 μg) at a 1:1 molar ratio. Reaction buffer supplemented with 10 μg/mL PDHc from porcine heart (Sigma) and reaction buffer with no additions were positive and negative controls, respectively. OD_600_ values were measured with a Multiskan Spectrophotometer (Thermo Fisher Scientific) for 30 min at 3-min intervals.

### Mouse polyclonal antisera against recombinant proteins and *M*. *gallisepticum* whole cells

Purified rPDHA and rPDHB proteins, and *M*. *gallisepticum* whole cells (inactivated with 0.4% formalin for 16 h at 37°C) were emulsified with an equal volume of Freund’s complete adjuvant (Sigma) and used to immunize 6-week-old female BALB/c mice (SLAC, Shanghai, China) via multipoint subcutaneous injection respectively (100 μg purified protein or 10^10^ colony forming units [CFUs] *M*. *gallisepticum* whole cells per mouse). After the first immunization, two boosters were given at 2-week intervals. Two non-immunized mice were used as negative controls. Tail vein blood from immunized and non-immunized mice was collected and the titres of polyclonal antisera measured by indirect enzyme-linked immunosorbent assay (iELISA) as previously described [22], with 96-well plates coated with coating buffer (16 mM Na_2_CO_3_, 34 mM NaHCO_3_, pH 9.6) containing purified protein (0.5 μg per well) or *M*. *gallisepticum* total protein prepared by sonic disruption of *M*. *gallisepticum* bacteria (0.5 μg per well) overnight at 4°C. When titres significantly increased, blood samples were collected from the infraorbital sinuses of mice and serum samples were separated and stored at −20°C.

### Surface localization, distribution and immunogenicity analyses

Surface localization of rPDHA and rPDHB on *M*. *gallisepticum* cells was determined using suspension immunofluorescence assays. *M*. *gallisepticum* strain R_*low*_ cells were collected at mid-logarithmic phase by centrifugation and washed three times with phosphate buffer saline (PBS, 137 mM NaCl, 2.7 mM KCl, 10 mM Na_2_HPO_4_, 2 mM KH_2_PO_4_; pH 7.4). Mycoplasma pellets were resuspended in PBS buffer containing 5% (w/v) skim milk and incubated for 1 h at 37°C. After centrifugation, cells were re-suspended in mouse anti-rPDHA (1:100) or anti-rPDHB serum (1:100) and incubated for 1 h at 37°C. Cells were washed three times with centrifugation and incubated with fluorescein isothiocyanate (FITC)-conjugated goat anti-mouse IgG (Sigma; 1:200) for 1 h at 4°C. After washing three times, pellets were re-suspended in PBS, spread onto glass slides, and observed by fluorescence microscope (Ti-S; Nikon, Tokyo, Japan). Mouse antiserum against *M*. *gallisepticum* rEno was used as positive control [22]. We previously found that *M*. *synoviae* fructose-bisphosphate aldolase (FBA) is a cytoplasmic protein and is not on the membrane surface [40]. *M*. *gallisepticum* FBA had the same distribution in *M*. *gallisepticum* cells as in *M*. *synoviae* (data not shown). Therefore, mouse antiserum against *M*. *gallisepticum* recombinant FBA (rFBA) and non-immunized mouse serum were used as negative controls.

To detect the subcellular localization of PDHA and PDHB in *M*. *gallisepticum*, membrane and cytoplasmic protein fractions of *M*. *gallisepticum* were extracted using ReadyPrep protein extraction kits (Membrane I; Bio-Rad, Hercules, CA, USA) according to the manufacturer’s instruction. Total cell proteins were prepared by sonic disruption of *M*. *gallisepticum* bacteria using an ultrasonic cell disruptor (Jingxin, Shanghai, China). Proteins were quantified using BCA protein assay kits (Pierce). Western blots were performed using 8 μg total cell proteins, membrane proteins, or cytoplasmic proteins as previously described [18]. Proteins were separated by SDS-PAGE and transferred to nitrocellulose (NC) membranes (Bio-rad). NC membranes were incubated with mouse anti-rPDHA or anti-rPDHB serum at 1:1,000 dilution. After washing three times with PBST (PBS buffer containing 0.05% Tween-20), NC membranes were incubated in horseradish peroxidase (HRP)-conjugated goat anti-mouse immunoglobulin G (IgG; 1:8,000; Sigma-Aldrich) and visualized with chemiluminescence (ECL) substrate kits (Thermo Fisher). Mouse anti-rEno or anti-rFBA sera were used as controls.

Western blot and ELISA were used for immunogenicity analyses of rPDHA and rPDHB. Purified rPDHA or rPDHB at 1, 0.5, 0.25, or 0.125 μg per lane were analysed by Western blot as described above. NC membranes with transferred proteins were incubated with *M*. *gallisepticum*-infected chicken serum (1:500) or *M*. *gallisepticum*-negative chicken serum (1:500, negative control). After washing, NC membranes were incubated with HRP-conjugated goat anti-chicken IgY (1:8,000; Abbkine, Redlands, CA, USA) and visualised with ECL substrate kits (Thermo Fisher). In an ELISA assay, 96-well ELISA plates (Corning) were coated with *M*. *gallisepticum* total proteins (0.3 μg per well) and blocked with 5% skim milk. Mouse anti-rPDHA and anti-rPDHB serum were diluted to the same titres, and subjected to further serial dilution to 1:200, 1:400, 1:800, 1:1,600, and 1:3,200. After adding the diluted antiserum to the plates and incubated for 2 h at 37°C, the plates were washed and HRP-conjugated goat anti-mouse Ig G (1:5,000; Sigma-Aldrich) was added for 1 h incubation at 37°C. Plates were incubated with 3,3',5,5'-tetramethylbenzidine (TMB) substrate solution (Tiangen) for 10 min and reactions were stopped with 2 M H_2_SO_4_. Absorbance at OD_450_ was measured with a spectrophotometer (Thermo Fisher Scientific). All experiments were done in triplicate.

### Complement-dependent bactericidal assays

Bactericidal assays were performed using mouse anti-rPDHA or anti-rPDHB serum as previously described [23]. Each antiserum was diluted to an ELISA titre of 1:5,000, 40 μL of anti-rPDHA or anti-rPDHB mouse serum, or a combination of each 20 μL anti-rPDHA and anti-rPDHB mouse serum were added with 120 μL *M*. *gallisepticum* suspension (5×10^3^ CFU mL^−1^) and 40 μL complement serum (CVCC; 1:5), and incubated for 1 h at 37°C. Subsequently, each 100 μL reaction mixture was spread onto mycoplasma agar plates in 60-mm dishes and cultured for colony counting. Mouse antiserum against *M*. *gallisepticum* whole cells was the positive control and non-immunized mouse serum was the negative control. Experiments were independently repeated three times. All mouse serum samples were inactivated by incubation for 30 min at 56°C before use. Bactericidal percentages were calculated as: (1—CFU from antiserum treatment / CFU from non-immunized serum treatment) × 100%.

### Indirect immunofluorescence and inhibition assays

To detect adherence of rPDHA and rPDHB to DF-1 cells, indirect immunofluorescence assays were performed. DF-1 cells were propagated on glass coverslips in 6-well cell culture plates (Corning) in DMEM with 10% FBS for 24 h. DF-1 cell monolayers were washed three times with PBS and incubated with rPDHA or rPDHB at 10 μg per well in DMEM for 1 h at 37°C with 5% CO_2_ for adhesion assays. DMEM without addition of recombinant protein was used as a negative control. For inhibition tests, each 10 μg rPDHA or rPDHB in DMEM was pre-incubated with respective mouse anti-rPDHA serum (1:100) or anti-rPDHB serum (1:100) at 37°C for 1 h. The rPDHA and rPDHB treated with non-immunized mouse serum were used as negative controls. After incubation with proteins, cells were washed five times with PBS-1% BSA, fixed with 4% paraformaldehyde (pH 7.4, Sigma) for 20 min at room temperature and blocked with 1% (w/v) bovine serum albumin (BSA) in PBS for 2 h at 37°C. Mouse anti-rPDHA or anti-rPDHB serum was diluted (1:1,000) in PBS-1% BSA buffer and added to the dishes for overnight incubation at 4°C. After washing three times, dishes were overlaid with goat anti-mouse IgG (H+L)-DyLight 488 (Abbkine, 1:400) at 37°C for 1 h and 10 μM 1,1'-dioctadecyl-3,3,3',3'-tetramethylindocarbocyanine perchlorate (DilC18(3); Beyotime, Shanghai, China) was used to label cell membranes at room temperature for 10 min. To label cell nuclei, 0.1 μg/mL 4',6-diamidino-2-phenylindole (DAPI; Beyotime) was added at room temperature for 10 min. Cells were observed by laser scanning confocal microscope (LSM800; Zeiss, Oberkochen, German). All experiments were repeated in triplicate.

### Surface display assays

To examine the effects of *M*. *gallisepticum* PDHA and PDHB on bacterial adhesion, we used a previously constructed surface display system for pIGN that contained InaZN as the anchoring motif and EGFP as the reporter [38]. With the pIGN system, the protein was expressed as a fusion with InaZN and EGFP, and displayed on the surface of *E*. *coli* BL21 (DE3) cells. This system was demonstrated for the detection of mycoplasma adhesion proteins. Full-length *pdhA* and *pdhB* gene fragments were inserted into pIGN at the *Bam*H I/*Xho* I and *Bam*H I/*Eco*R I sites, respectively, and fusion proteins were expressed in *E*. *coli* BL21 (DE3) with 1 mM IPTG for 12 h at 37°C. For adhesion assays, induced *E*. *coli* BL21 (DE3) cells containing pIGN-PDHA or pIGN-PDHB were washed with PBS, re-suspended in DMEM, and used to infect a monolayer of DF-1 cells for 2 h at 37°C at 50 MOI. After removing non-adherent *E*. *coli* cells by washing with PBS, cells were fixed with 4% paraformaldehyde for 15 min and stained with DilC18(3) (Beyotime) and DAPI (Beyotime) as described above. Induced *E*. *coli* BL21 (DE3) cells harbouring pIGN were used as negative controls. For adhesion inhibition assays, induced *E*. *coli* BL21 (DE3) cells containing pIGN-PDHA or pIGN-PDHB were pre-incubated with mouse antisera against rPDHA or rPDHB for 1 h at 37°C before adding to DF-1 cells. *E*. *coli* BL21 (DE3) cells treated with non-immunized rabbit serum were used as negative controls. After staining with DilC18 (3) and DAPI, cells were observed using a fluorescence microscope (Ti-S; Nikon). All experiments were repeated in triplicate.

### Adherence inhibition assay on colony counting

Inhibition of *M*. *gallisepticum* adhesion to DF-1 cells by mouse anti-rPDHA or anti-rPDHB serum was identified by colony counting assays in plates as previously described, with some modifications [22]. *M*. *gallisepticum* strain R_*low*_ at mid-logarithmic phase was collected by centrifugation at 4000 ×*g* for 10 min at 4°C, and treated with 100 μL mouse anti-rPDHA or anti-rPDHB serum at the same antibody titre (1:500), or a combination of each 50 μL mouse antiserum (1:500 antibody titre) for 1 h at 37°C. Mouse anti*-M*. *gallisepticum* serum and non-immunized mouse serum were used as positive and negative controls. Monolayer DF-1 cells in 24-well plates (Corning) were washed three times with PBS and infected for 2 h with serum*-*pre-treated *M*. *gallisepticum* bacteria at 200 MOI. *M*. *gallisepticum* treated with non-immunized serum was used as the negative control. After infection, cells were washed three times with PBS and dissociated with 0.05% trypsin for 10 min at 37°C. Cell lysates were serially diluted and plated onto mycoplasma solid medium for 5–7 days at 37°C with 5% CO_2_. Mycoplasma colonies were counted and inhibition of mycoplasma adherence to DF-1 cells by antisera was calculated as: (1- CFU from antiserum treatment/CFU from non-immunized serum treatment) × 100%. All experiments were done in triplicate.

### Binding of rPDHA and rPDHB to chicken plasminogen

To test the binding activity of rPDHA and rPDHB to chicken plasminogen (cPlg), Western blots and ELISA assays were conducted as previously described [18].

For Western blots, 4 μg of *M*. *gallisepticum* total proteins, purified rPDHA, and rPDHB were subjected to SDS-PAGE respectively and transferred to NC membranes. Membranes were blocked with 5% skim milk in PBST at room temperature for 40 min and washed three times with PBST. Membranes were incubated with 10 μg/mL cPlg (Cell Sciences, Canton, MA, USA) in PBST and stored for 2 h at 37°C. Membranes not incubated with cPlg were used as negative controls. After washing, membranes were incubated with rabbit anti-cPlg IgG fraction polyclonal antibody (1:1000; Cell Sciences) for 2 h at 37°C. After additional washing, membranes were incubated with goat anti-rabbit IgG-HRP (1:8000; Sigma-Aldrich) for 1 h at 37°C and visualized with an ECL substrate kit (Thermo Fisher).

For ELISA assays, ELISA plates were coated with 1 μg per well purified rPDHA or rPDHB protein, or a mixture of rPDHA and rPDHB in equal quantities of 0.5 μg per well and incubated overnight at 4°C. *M*. *gallisepticum* total proteins (1 μg per well) and BSA (1 μg per well) were used as positive and negative controls respectively. After washing three times with PBST, wells were blocked with PBST containing 5% skim milk for 2 h at 37°C. Serially diluted cPlg (0, 0.015, 0.03, 0.06, 0.125, 0.25, 0.5 μg or 1.0 μg per well) in PBST was added to wells and incubated for 2 h at 37°C. After washing, plates were incubated with rabbit anti-cPlg polyclonal antibody (1:2000 for 1.5 h; Cell Sciences) at 37°C. After washing, plates were incubated with HRP-conjugated goat anti-rabbit IgG (1:5,000; Sigma-Aldrich) for 1 h at 37°C. Plates were incubated with TMB substrate solution (Tiangen) and the absorbance at OD_450_ was measured. All experiments were done in triplicate.

### Statistical analysis

Data are expressed as means ± SD for adhesion and adhesion inhibition assays. Student’s *t-*test and two-way ANOVA were performed with the software package in GraphPad Prism version 6 (La Jolla, CA, USA). Differences were considered statistically significant at *p* < 0.05 or very significant at *p* < 0.01, *p* < 0.001 or *p* < 0.0001.

## Results

### Expression, purification and antibody production of *M*. *gallisepticum* rPDHA and rPDHB

**Fig 1:**
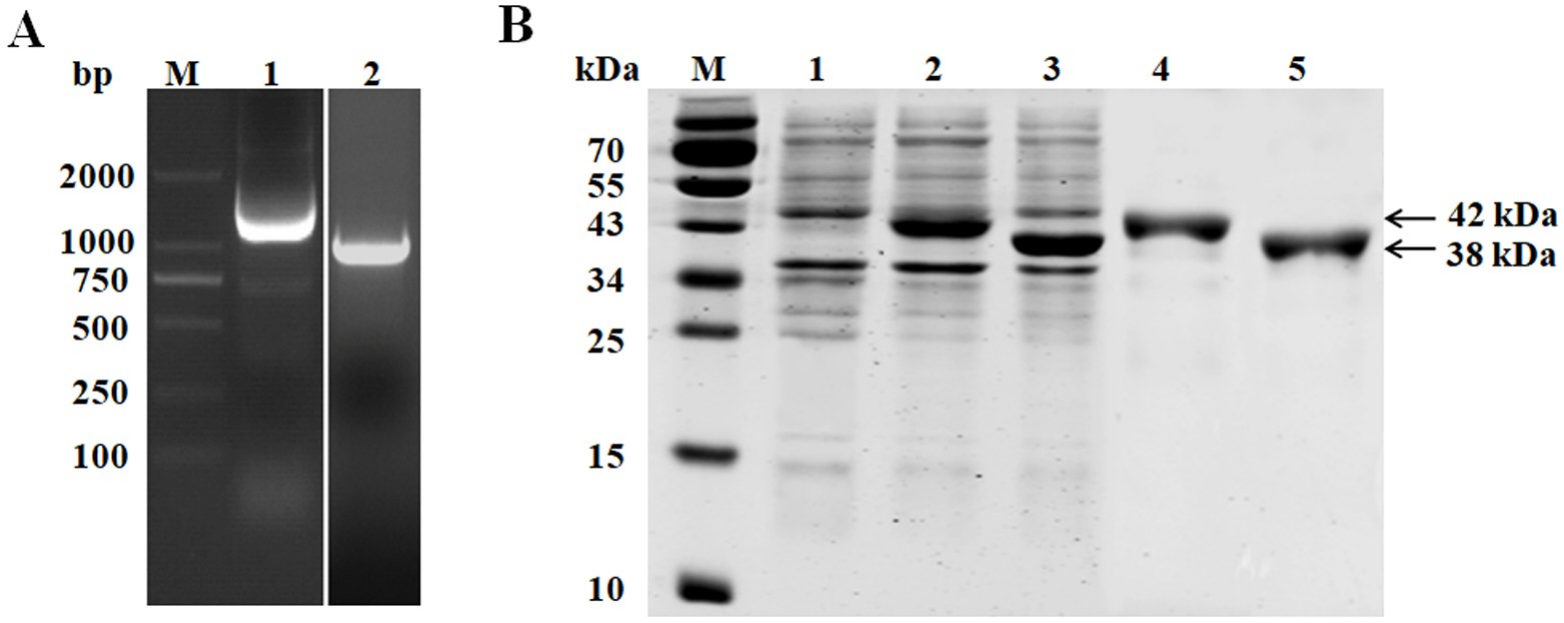
Cloning and expression of the *M*. *gallisepticum pdhA* and *pdhB* genes. (A) Results of PCR amplification. Lane M: DNA marker; Lane 1: *pdhA* gene of *M*. *gallisepticum*; Lane 2: *pdhB* gene of *M*. *gallisepticum*. (B) SDS-PAGE of rPDHA and rPDHB. Lane M: Prestained protein marker; Lane 1: *E*. *coli* BL21 (DE3) transformed with pET-28a (+) induced by IPTG (negative control); Lane 2: *E*. *coli* BL21 (DE3) containing recombinant pET28a-PDHA induced by IPTG; Lane 3: *E*. *coli* BL21 (DE3) containing recombinant pET28a-PDHB induced by IPTG; Lane 4: Purified rPDHA protein (42 kDa); Lane 5: Purified rPDHB protein (38 kDa).

Full-length *pdhA* (1080 bp) and *pdhB* (978 bp) gene fragments were obtained from overlapping PCR amplification (Fig 1A), cloned into pET-28a (+) and transformed into *E*. *coli* BL21 (DE3) cells. IPTG was used to induce expression of rPDHA and rPDHB, which were subjected to SDS-PAGE and showed approximate molecular masses of 42 kDa for rPDHA and 38 kDa for rPDHB (Fig 1B, lanes 2 and 3). Purified rPDHA and rPDHB proteins were also obtained (Fig 1B, lanes 4 and 5). After three immunizations, mouse antisera against rPDHA or rPDHB were collected and the antibody titres were determined as 1:51,200 for rPDHA and 1:204,800 for rPDHB in an ELISA assay.

### Enzymatic activities of rPDHA and rPDHB

**Fig 2:**
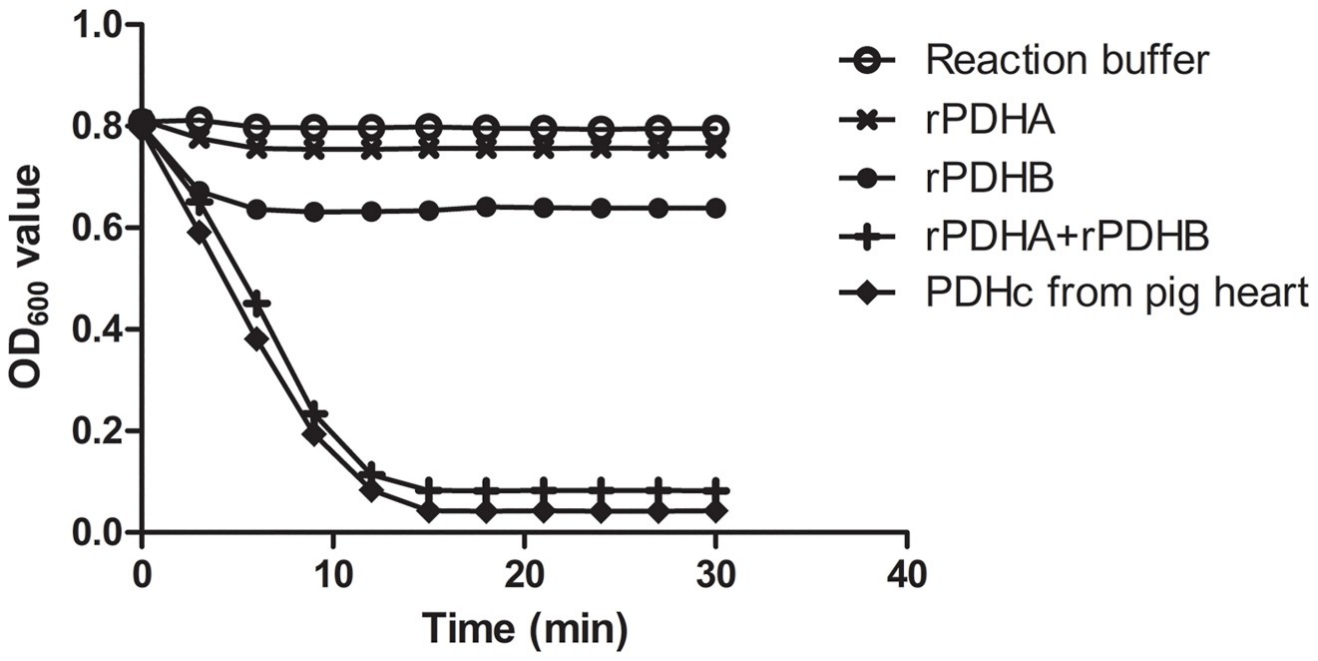
Enzymatic activity assay. Enzymatic activities of purified rPDHA and rPDHB measured by reduction of 2,6-DCPIP at OD_600_. Equimolar mixtures of rPDHA and rPDHB had similar enzymatic activity as the positive control, porcine heart PDHc. The rPDHB had weak enzymatic activity while rPDHA had no detectable enzymatic activity, similar to the negative control.

The enzymatic activities of rPDHA and rPDHB were measured by detecting reduction of 2,6-DCPIP at OD_600_. The rPDHA, rPDHB and a mixture of rPDHA and rPDHB at a 1:1 molar ratio were tested for enzymatic activity. PDHc from porcine heart and reaction buffer without additions were the positive and negative controls, respectively. Alone, rPDHA had no detectable catalytic activity and rPDHB displayed weak catalytic activity. High activity was noted when rPDHA was mixed with rPDHB at a 1:1 molar ratio, similar to the positive control porcine heart PDHc (Fig 2).

### Surface localization, distribution and immunogenicity analyses of *M*. *gallisepticum* PDHA and PDHB

**Fig 3:**
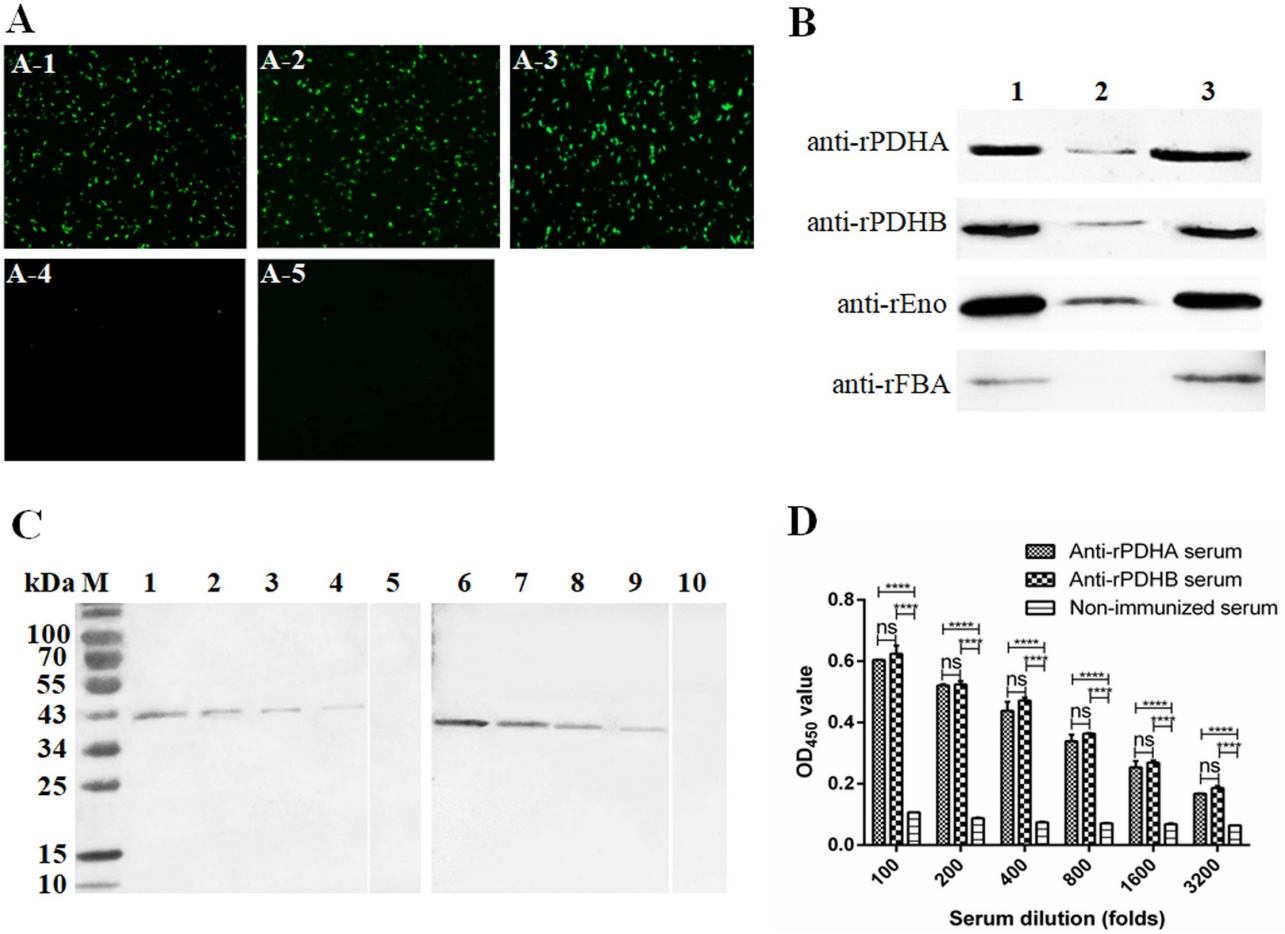
Surface localization, distribution and immunogenicity. (A) Suspension immunofluorescence assays of *M*. *gallisepticum* using mouse antisera and FITC-conjugated goat anti-mouse antibody. A-1: mouse antiserum against rPDHA; A-2: mouse antiserum against rPDHB; A-3: mouse antiserum against rEno (positive control); A-4: mouse antiserum against rFBA (negative control); A-5: non-immunized mouse serum (negative control). FITC-stained *M*. *gallisepticum* indicated that PDHA and PDHB were surface-exposed (400 magnification). Mouse anti-rEno serum (A-3) was the positive control, mouse anti-rFBA serum (A-4) and non-immunized mouse serum (A-5) were negative controls. (B) Subcellular localization of PDHA and PDHB in *M*. *gallisepticum* by Western blots. Total cell proteins (lane 1), membrane component (lane 2) and cytoplasmic component (lane 3) of *M*. *gallisepticum* were incubated with mouse anti-rPDHA (1:1,000) or anti-rPDHB serum (1:1,000). Mouse anti-rEno serum and anti-rFBA serum were controls. (C) Immunogenicity analysis by Western blots. Serially diluted, purified rPDHA protein (1, 0.5, 0.25, and 0.125 μg for lane 1, 2, 3 and 4) and rPDHB protein (1, 0.5, 0.25, 0.125 μg for lane 6, 7, 8, and 9) were blotted by *M*. *gallisepticum*-infected chicken serum at a dose-dependent pattern; Purified rPDHA (lane 5) and rPDHB (lane 10) showed no band with *M*. *gallisepticum*-negative chicken serum. (D) Immunogenicity analysis by an ELISA assay. Mouse anti-rPDHA and anti-rPDHB serum were diluted to the same antibody titre (1:51,200), and subjected to further serial dilution to 1:200, 1:400, 1:800, 1:1,600, and 1:3,200. *M*. *gallisepticum* total proteins (0.3 μg per well) coated plates showed positive binding with mouse anti-rPDHA and anti-rPDHB serum but not non-immunized mouse serum. Statistical analyses were by two-way ANOVA in GraphPad Prism version 6 (****, *p*< 0.0001; ns, no significant difference).

**Fig 4:**
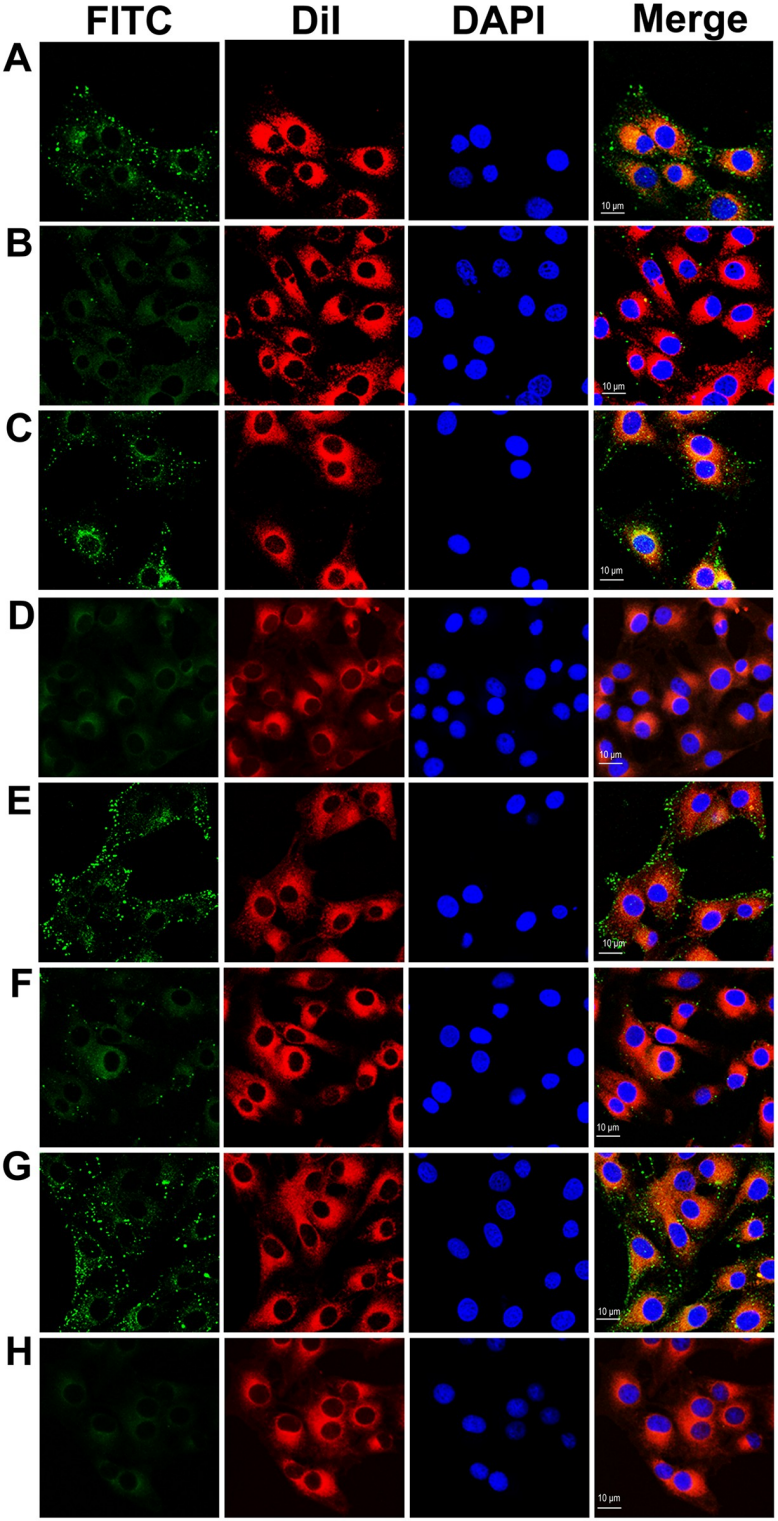
Indirect immunofluorescence assays. (A–D) Adhesion and inhibition of rPDHA to DF-1 cells. Adhesion of rPDHA protein to DF-1 cells (A). Inhibition by mouse anti-rPDHA serum (B). No inhibition with non-immunized mouse serum (C). No signals with DMEM detected by mouse anti-rPDHA serum (D). (E–H) Adhesion and inhibition of rPDHB to DF-1 cells. Adhesion of rPDHB protein to DF-1 cells (E). Inhibition by mouse anti-rPDHB serum (F). No inhibition with non-immunized mouse serum (G). No signals with DMEM detected by mouse anti-rPDHB serum (H). Column FITC, rPDHA or rPDHB protein labelled with antisera and goat anti-mouse FITC conjugate. Column Dil, cell membrane labelled using DilC18(3). Column DAPI, cell nuclei labelled by DAPI; Column merge, merge of fluorescent images.

**Fig 5:**
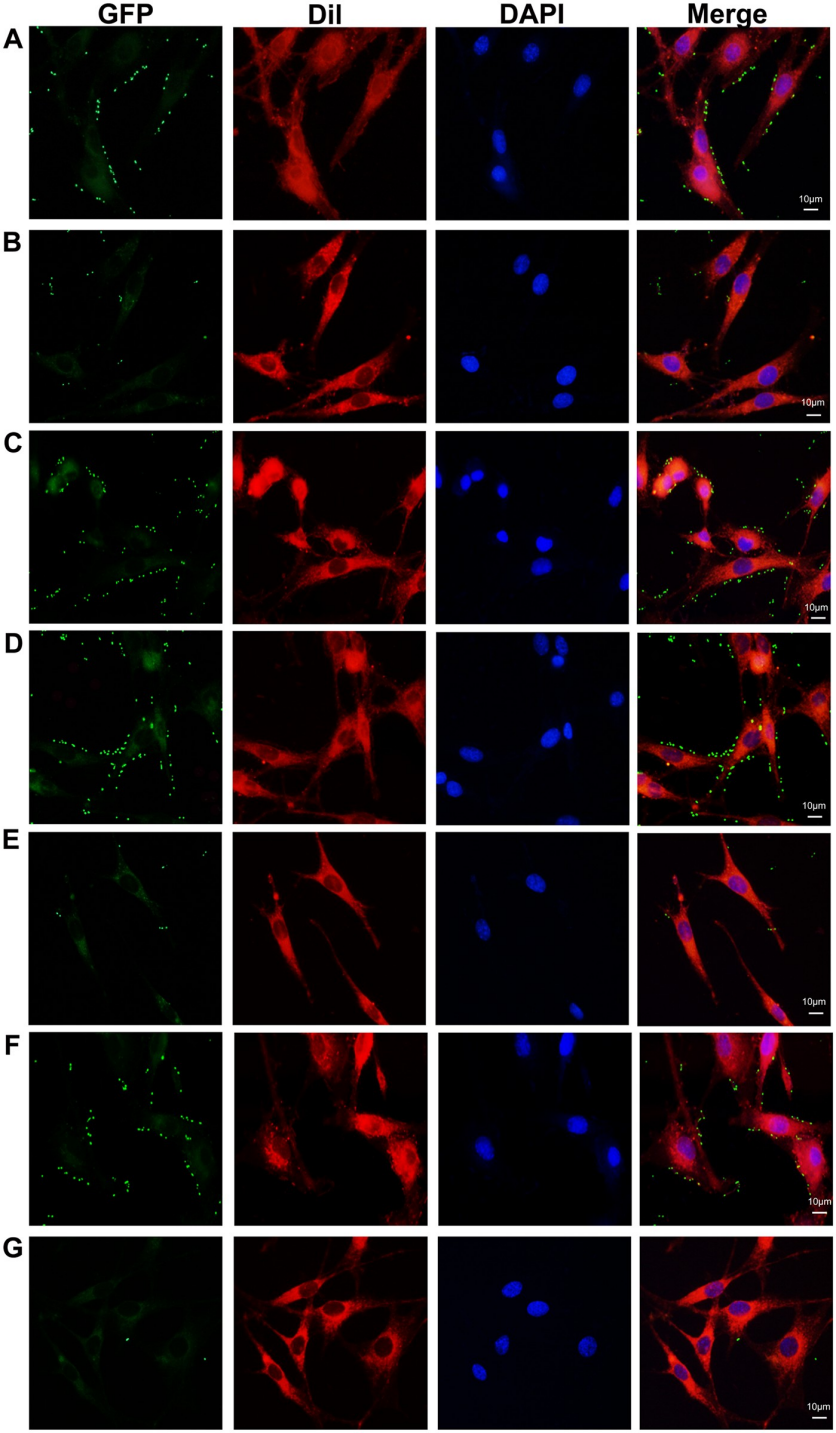
Surface display assays. Adhesion and inhibition of *E*. *coli* BL21 (DE3) containing pIGN-PDHA or pIGN-PDHB to DF-1 cells. Adhesion of *E*. *coli* BL21 (DE3) containing pIGN-PDHA (A) or pIGN-PDHB (D) to DF-1 cells. Adhesion of *E*. *coli* BL21 (DE3) containing pIGN-PDHA to DF-1 cells inhibited by mouse anti-rPDHA (B) but not non-immunized mouse serum (C). Adhesion of *E*. *coli* BL21 (DE3) containing pIGN-PDHB to DF-1 cells inhibited by mouse anti-rPDHB (E) but not non-immunized mouse serum (F). PIGN (G) was used as the negative control. Column GFP, recombinant *E*. *coli* cells containing GFP fusion protein. Column Dil, cell membrane labelled by DilC18(3). Column DAPI, cell nuclei labelled by DAPI; Column merge, merge of fluorescent images.

The surface localization of PDHA and PDHB in *M*. *gallisepticum* was determined by suspension immunofluorescence assays using mouse anti-rPDHA and anti-rPDHB sera and fluorescein isothiocyanate (FITC)-conjugated goat anti-mouse IgG (Sigma-Aldrich). *M*. *gallisepticum* cells incubated with mouse anti-rPDHA (Fig 3A-1), anti-rPDHB (Fig 3A-2) or anti-rEno (Fig 3A-3) sera were stained with FITC, whereas *M*. *gallisepticum* cells incubated with mouse anti-rFBA serum (Fig 3A-4) or non-immunized mouse serum (Fig 3A-5) exhibited no fluorescence. The results suggested that both the *M*. *gallisepticum* PDHA and PDHB were surface exposed.

The distribution of PDHA and PDHB in *M*. *gallisepticum* was determined by Western blots. PDHA and PDHB were mainly distributed in the cytoplasmic component of *M*. *gallisepticum*, with a small amount in the membrane component (Fig 3B). The results suggested that pyruvate dehydrogenase E1 mainly functions as a biological enzyme in the cytoplasm and may have other functions as a surface-displayed protein. In addition, the positive control Eno was distributed in both the cytoplasmic and membrane components of *M*. *gallisepticum*. The negative control FBA was only in the cytoplasmic component of *M*. *gallisepticum*, confirming no cytoplasmic protein contamination in the membrane proteins.

Immunogenicity analysis of rPDHA and rPDHB was determined by Western blot and ELISA assays. Western blot analyses with *M*. *gallisepticum*-infected chicken sera showed positive bands for all detected concentrations of rPDHA (Fig 3C, lanes 1 to 4) and rPDHB (Fig 3C, lanes 6 to 9) with band densities in a dose-dependent pattern. No band was present after interaction with *M*. *gallisepticum*-negative chicken serum. In an ELISA assay, mouse anti-rPDHA or anti-rPDHB sera were diluted to the same antibody titre of 1:51,200 and then subjected to two-fold serial dilution for immunogenicity analyses. Anti-rPDHA and anti-rPDHB mouse sera with the same antibody titre had similar reactivity against *M*. *gallisepticum* whole antigen (Fig 3D). These results suggested that both *M*. *gallisepticum* PDHA and PDHB were immunogenic proteins.

### Antisera bactericidal assays

To evaluate the protective abilities of *M*. *gallisepticum* PDHA and PDHB, we performed complement-dependent antisera bactericidal assays. As shown in Table 2, the positive control of mouse anti-*M*. *gallisepticum* serum had a bactericidal rate of 91.0% (Table 2), mouse anti-rPDHA serum alone had a bactericidal rate of 48.0% and anti-rPDHB alone had a rate of 75.1%. A combination of rPDHA and rPDHB antisera had a bactericidal rate of 65.2%, similar to the means of 48.0% and 75.1%, respectively. Compared with non-immunized mouse serum, both mouse anti-rPDHA and anti-rPDHB sera showed significant bactericidal activity (*p*< 0.001).

**Table 2:** Bactericidal rates of mouse antisera against rPDHA or rPDHB.

Sera	Mean CFU ± SD	Bactericidal rates ^b^ (%)
Anti-rPDHA serum	128.5 ± 10.0 ^a^	48.0*** ^b^
Anti-rPDHB serum	61.7 ± 9.1 ^a^	75.1*** ^b^
Combination of rPDHA and rPDHB antisera	86.1± 6.7 ^a^	65.2*** ^b^
Anti-*M*. *gallisepticum* serum	22.3 ± 6.5 ^a^	91.0*** ^b^
Non-immunized serum	247.3 ± 12.6 ^a^	-

^a^ Results were from three replicate experiments.

^b^ Statistical significance compared to non-immunized serum determined by Student’s *t*-test (***, *p*< 0.001).

### Adherence of rPDHA and rPDHB to DF-1 cells by indirect immunofluorescence assays

Adhesion of rPDHA and rPDHB to DF-1 cells was detected by indirect immunofluorescence assays. Both rPDHA (Fig 4A) and rPDHB (Fig 4E) adhered to DF-1 cells and adherence was effectively inhibited by the corresponding mouse anti-rPDHA or anti-rPDHB sera (Fig 4B and 4F). Negative serum from non-immunized mice did not affect adherence of rPDHA and rPDHB to DF-1 cells (Fig 4C and 4G) and cells incubated with antiserum only showed no green fluorescence (Fig 4D and 4H). These results indicated that *M*. *gallisepticum* PDHA and PDHB were potential adhesion-related proteins.

### Surface display system indicated rPDHA and rPDHB adhesion abilities

Recombinant *E*. *coli* BL21 (DE3) containing the vector of pIGN, pIGN-PDHA, or pIGN-PDHB were induced and identified on SDS-PAGE gels. Fusion proteins of InaZN-EGFP (46 kDa), InaZN-EGFP-PDHA (86 kDa), and InaZN-EGFP-PDHB (81 kDa) were expressed in *E*. *coli* (S1 Fig). In adhesion and inhibition assays, recombinant *E*. *coli* containing pIGN-PDHA or pIGN-PDHB adhered to DF-1 cells (Fig 5A and 5D), and adherence of recombinant *E*. *coli* was significantly restrained by mouse antiserum against rPDHA or rPDHB (Fig 5B and 5E). *E*. *coli* containing pIGN showed no adhesion ability (Fig 5G). These results suggested that *M*. *gallisepticum* PDHA and PDHB mediated adhesion of bacteria to host cells.

### Adherence inhibition of *M*. *gallisepticum* to DF-1 cells by mouse antisera against rPDHA or rPDHB

Adherence inhibition assays were performed using *Mycoplasma*-free DF-1 cells. *M*. *gallisepticum* pre-treated with mouse anti-*M*. *gallisepticum* serum or non-immunized mouse serum were used as controls. Adherence inhibition rates (%) by mouse anti-rPDHA or mouse anti-rPDHB serum of *M*. *gallisepticum* adhering to DF-1 cells were 30.2% (*p* < 0.001) and 45.1% (*p* <0.001) respectively (Table 3). The adherence inhibition rate of a combination of rPDHA and rPDHB antisera (1:250 antibody titre of each serum) was 72.5% (*p*< 0.001), which was higher than that of rPDHA or rPDHB antiserum treatment alone. This result suggested synergistic effects of rPDHA and rPDHB antisera on adherence inhibition capacity.

**Table 3:** Adherence inhibition rates of the antisera.

Sera	Mean CFU (×10)± SD	Inhibition rates (%)
Anti-rPDHA serum	443.3 ± 38.6 ^a^	30.2*** ^b^
Anti-rPDHB serum	348.7 ± 45.1 ^a^	45.1*** ^b^
Combination of rPDHA and rPDHB antisera	174.5 ± 31.9 ^a^	72.5*** ^b^
Anti-*M*. *gallisepticum* serum	100.7 ± 16.7 ^a^	84.1*** ^b^
Non-immunized serum	635.0 ± 59.8 ^a^	-

^a^ Data were from triplicate experiments.

^b^ Compared to non-immunized serum by Student’s *t*-test (***, *p*< 0.001).

### Binding activity of rPDHA and rPDHB to cPlg

**Fig 6:**
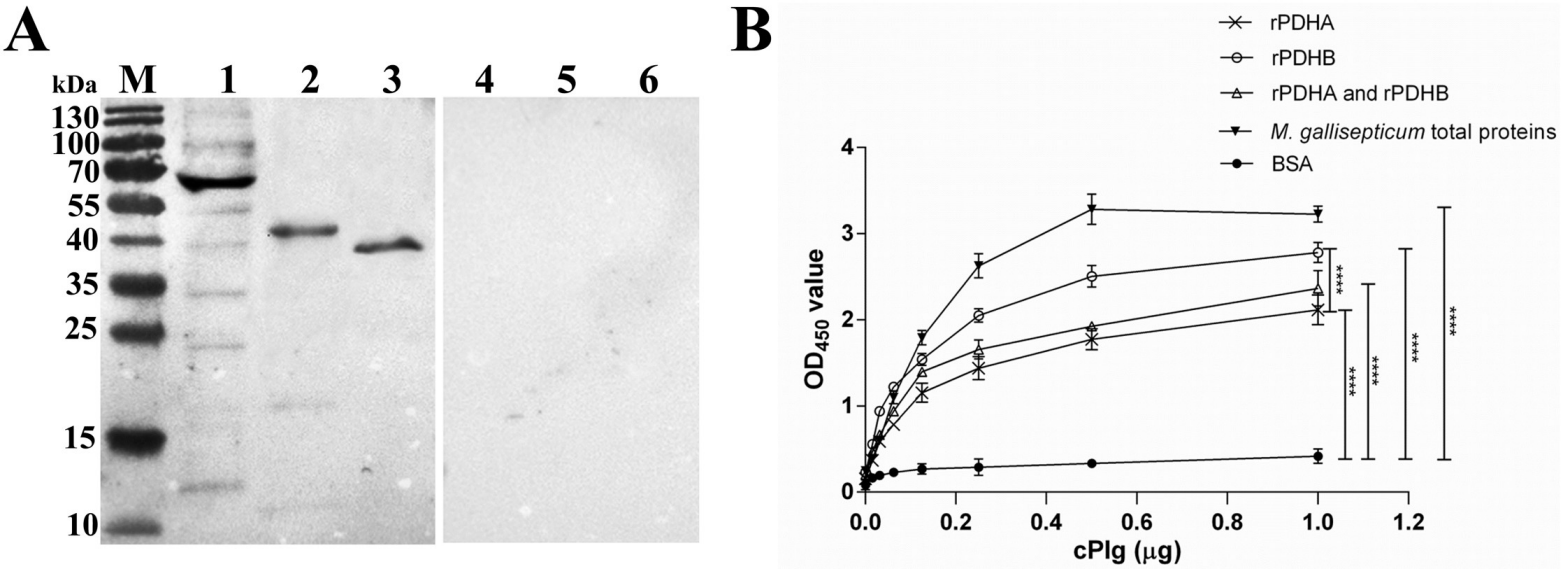
Binding assays of rPDHA or rPDHB with cPlg. (A) Binding assays by Western blot using rabbit polyclonal antibody against cPlg. The total *M*. *gallisepticum* protein (lane 1), purified rPDHA (42 kDa; lanes 2 and 5) and rPDHB (38 kDa; lanes 3 and 6) incubated with 10 μg/mL cPlg and rabbit anti-cPlg (lanes 1, 2 and 3) or rabbit anti-cPlg alone (lanes 4, 5 and 6, negative control). Both rPDHA and rPDHB bound cPlg (lanes 2 and 3), but neither bound rabbit anti-cPlg (lanes 5 and 6). (B) Binding assay by ELISA. ELISA plates were coated with purified rPDHA or rPDHB at 1 μg per well, or a mixture of rPDHA and rPDHB at 0.5 μg per well each, incubated with serially diluted cPlg (0, 0.015, 0.03, 0.06, 0.125, 0.25, 0.5 or 1.0 μg per well) and probed with rabbit polyclonal antibody against cPlg. Plates coated with *M*. *gallisepticum* total proteins or BSA (1 μg per well) were positive and negative controls. Statistical analyses were by two-way ANOVA in GraphPad Prism version 6 (****, *p*< 0.0001).

Western blots demonstrated that *M*. *gallisepticum* total proteins, purified rPDHA, and rPDHB (Fig 6A, lanes 1, 2 and 3) interacted with cPlg but not with the rabbit polyclonal antibody against cPlg alone (Fig 6A, lanes 4, 5 and 6). In addition, there were many positive bands including the bands for PDHA (39 kDa) and PDHB (34 kDa) when *M*. *gallisepticum* total proteins interacted with cPlg. In an ELISA binding assays (Fig 6B), OD_450_ values from coating with *M*. *gallisepticum* total proteins, rPDHA, rPDHB, or a mixture of rPDHA and rPDHB were significantly higher than that from coating with BSA (*p* < 0.0001). This result indicated that *M*. *gallisepticum* total proteins, rPDHA and rPDHB bound to cPlg in a dose-dependent manner. In addition, the binding ability of rPDHB was significantly higher than that of rPDHA (*p*< 0.0001). The mixture of rPDHA and rPDHB showed moderate Plg-binding ability compared with rPDHA or rPDHB alone.

## Discussion

Due to limited genome sizes, mycoplasmas possess limited biosynthetic and metabolic capabilities and are parasites, using infected host cells for their nutrition [41, 42]. To use limited genomic resources more effectively, many mycoplasma glycolytic enzymes are typically expressed on the surface. They function as multifunctional enzymes, in adhesion to host epithelia, binding to host components, colonization, persistence and invasion of the host tissues [24, 43]. PDHA and PDHB of *M*. *gallisepticum* were confirmed as surfaced-localized proteins, which makes it possible for them to participate in cytoadherence. However, most of the surface-exposed multifunctional enzymes including Eno, PDHA and PDHB, do not have typical signal peptides or membrane-anchoring mechanisms and are therefore described as “anchorless” or “non-classically secreted” proteins [44]. The mechanism of surface display for these enzymes remains unclear. A study showed that some cytoplasmic proteins including GroEL, DnaK, Eno, PDHB and PDHD are secreted in large amounts during the late stationary phase of *Bacillus subtilis*, and release of these proteins is not due to gross cell lysis but rather a process in which the protein domain structure is a contributing factor [44]. Another study indicated that *Streptococcus pneumoniae* Eno re-associates on the bacterial surface after secretion [45], suggesting a potential mechanism for its surface localization. However, whether *M*. *gallisepticum* PDHA or PDHB are secreted or re-associated on the bacterial cell-surface remains unknown. We collected supernatant proteins from *M*. *gallisepticum* liquid culture as described for *B*. *subtilis* with some modifications [44]. The supernatant was filtered (0.1 μm Millex; Millipore) to remove residual cells and concentrated by TCA-acetone precipitation. A mixture of secreted and medium proteins was obtained. Western blots with mouse anti-PDHA, anti-PDHB or anti-Eno serum showed no positive bands. Detecting secreted proteins from mycoplasma is not easy. A number of medium proteins (from horse serum, yeast extract or bouillon) are in *M*. *gallisepticum* supernatants from conventional cultures, which may make mycoplasma secreted proteins hard to be detected. Therefore, finding a low or no-protein medium that allows *M*. *gallisepticum* to grow is necessary, which needs to be further investigated.

Adhesion of mycoplasmas to host cell surfaces is a necessary stage for infection and parasitization. Through adhesion, mycoplasmas obtain essential nutrients such as carbohydrates, lipids and proteins from the host. Searching for adhesion-related proteins and elucidating their functions in cytoadherence are of great significance. In *M*. *pneumoniae*, PDHA and PDHB bind to HeLa [35] and A549 cells (a human lung carcinoma cell line) [36] in ELISA assays with HeLa cell-coated or A549 cell-coated plates. Adherence is significantly reduced by corresponding antisera. In *M*. *gallisepticum*, although some cytoadherence-associated proteins have been found, PDHA and PDHB have not been reported. In our study, adhesion assays were conducted by three different methods, indirect immunofluorescence assay, pIGN surface-displaying system, and colony counting assays. All methods confirmed that PDHA and PDHB were cytoadherence-associated proteins of *M*. *gallisepticum* and adherence was inhibited by corresponding antisera. We visualized the cytoadherence of PDHA and PDHB in *M*. *gallisepticum* using indirect immunofluorescence assays and a pIGN surface-displaying system. By these two methods, the negative controls of His-tagged FBA protein and *E*. *coli* containing pIGN (InaZ-EGFP) exhibited no adhesion, supporting the positive results for PDHA and PDHB. In colony counting assays, anti-rPDHA serum alone caused 30.2% of adhesion inhibition rates and anti-rPDHB serum alone caused 45.1%, indicating that both PDHA and PDHB might be involved in *M*. *gallisepticum* cytoadherence. Mixtures of PDHA and PDHB antisera caused higher adhesion inhibition rates of 72.5%, suggesting the combination of PDHA and PDHB antisera might have higher protective ability. In addition, disruption of *M*. *agalactiae* PDHB significantly reduces invasiveness in HeLa cells [46]. The absence of *M*. *agalactiae* PDHB influences initial colonization and systemic spreading of *M*. *agalactiae* during experimental infection of sheep [47], suggesting that PDHB may help microorganisms colonize and invade host cells and cross host tissue barriers. *M*. *gallisepticum* is confirmed to adhere to and invade cultured human epithelial cells (HeLa-229) and chicken embryo fibroblasts [48, 49]. Since cytoadherence is the first step for pathogens to invade host cells, *M*. *gallisepticum* PDHA and PDHB may also be involved in invasion into host cells. However, whether *M*. *gallisepticum* PDHA or PDHB is related to the pathogen invading the host remains to be further explored.

Host ECM proteins including Plg, Fn, β-actin, lactoferrin, laminin, and vitronectin were confirmed to be glycolytic enzyme-binding-related host proteins. Interactions of microbial proteins with host ECM proteins contributes to colonization [50–52] and dissemination [53, 54] and are important factors for virulence strategies [55]. Plg is a 92-kDa plasma protein and is the zymogen of plasmin, which is a serine protease that dissolves fibrin blood clots [56]. In *M*. *gallisepticum* and some other bacteria, Plg-binding is reported to markedly increase the cytoadherence to and invasion of host cells by bacteria [20, 49, 50]. Moonlighting proteins are hypothesized to help microorganisms cross host tissue barriers by binding to host cell Plg. Our study confirmed the binding ability of *M*. *gallisepticum* PDHA and PDHB to cPlg by both Western blot and ELISA. Whether Plg-binding is involved in cytoadherence and invasion of PDHA and PDHB to DF-1 cells, and whether PDHA and PDHB bind to other ECM proteins remain to be further explored. When *M*. *gallisepticum* total proteins interacted with cPlg, many positive bands appeared (Fig 6A, lane 1), implying many proteins in *M*. *gallisepticum* were Plg-binding proteins. In addition, a band between 55 and 70 kDa showed strong reaction with cPlg, while the PDHA (39 kDa) and PDHB (34 kDa) in *M*. *gallisepticum* showed weak band, which gave us the information that PDHA and PDHB may not be the major Plg-binding proteins in *M*. *gallisepticum*, and there are some other more important Plg-binding proteins need to be further investigated.

Multifunctional enzymes were confirmed as immunogenic proteins involved in modulating the host immune system. *Vibrio parahaemolyticus* Eno is reported to be a protective antigen [57] and FbaA and GAPDH of virulent pneumococci were identified as cross protective antigens, protecting mice from respiratory challenges [58]. Immunogenicity analysis showed that both rPDHA and rPDHB were immunogenic proteins in reactions with *M*. *galliseticum*-infected chicken serum. Complement-mediated bactericidal assays showed bactericidal rates of 48.0% for anti-rPDHA serum and 75.1% for anti-rPDHB serum, indicating that both PDHA and PDHB were protective antigens of *M*. *gallisepticum*. A combination of rPDHA and rPDHB antisera had a bactericidal rate of 65.2%, similar to the mean of 48.0% and 75.1%, suggesting that in combination, the two sera did not interfere with each other for bactericidal activity.

From prokaryotes to mammals, PDHc E1 typically contains two structural forms: one of two identical α subunits and another of four subunits of α2 and β2. Since the catalytic action relies on the combination of homodimers or heterotetramers, the phosphorylation or mutation of specific amino acid residues of subunits or disruption or deletion of PDHc E1 subunits is sufficient to inactivate the enzyme [39, 59]. In our study, rPDHA and rPDHB enzymatic activities were detected using 2,6-DCPIP assays as previously described [39]. For rPDHA or rPDHB alone, no or very weak catalytic activity was detected. However, when rPDHA and rPDHB were combined, strong enzymatic activity was observed. The results indicated that the two subunits of PDH E1 were indispensable and worked together.

In conclusion, we characterized the enzymatic activity of rPDHA and rPDHB, and found that both *M*. *gallisepticum* PDHA and PDHB were surface localized and immunogenic proteins. Furthermore, both PDHA and PDHB were identified as Plg-binding proteins involved in cytoadherence, suggesting they may be involved in bacterial colonization and dissemination in host cells and may contribute to mycoplasma virulence.

## Ethics approval and consent to participate

The animal experiments were performed in accordance with the Institutional Animal Care and Use Committee (IACUC) guidelines set by Shanghai Veterinary Research Institute, the Chinese Academy of Agricultural Sciences (CAAS). The experiments were approved by the Committee on the Ethics of Animal Experiments of Shanghai Veterinary Research Institute, CAAS (Permit Number: Shvri-mo-201708050680).

## Supporting information

S1 Fig SDS-PAGE of recombinant *E*. *coli* containing pIGN, pIGN-PDHA, and pIGN-PDHB. Lane 1: *E*. *coli* BL21 (DE3) transformed with pET-28a (+) induced by IPTG; Lane 2: *E*. *coli* BL21 (DE3) containing recombinant plasmid pIGN-PDHA induced by IPTG; Lane 3: *E*. *coli* BL21 (DE3) transformed with the pIGN-PDHB induced by IPTG. Lane 4: *E*. *coli* BL21 (DE3) transformed with pIGN induced by IPTG. Red marks, expressed recombinant fusion protein.
(TIF)
