# Supplementary figures and images for: Characterization of Mycoplasma gallisepticum pyruvate dehydrogenase alpha and beta subunits and their roles in cytoadherence
Source: PLoS One. 2018 Dec 10;13(12):e0208745. doi: 10.1371/journal.pone.0208745 (PMC6287819; doi:10.1371/journal.pone.0208745)

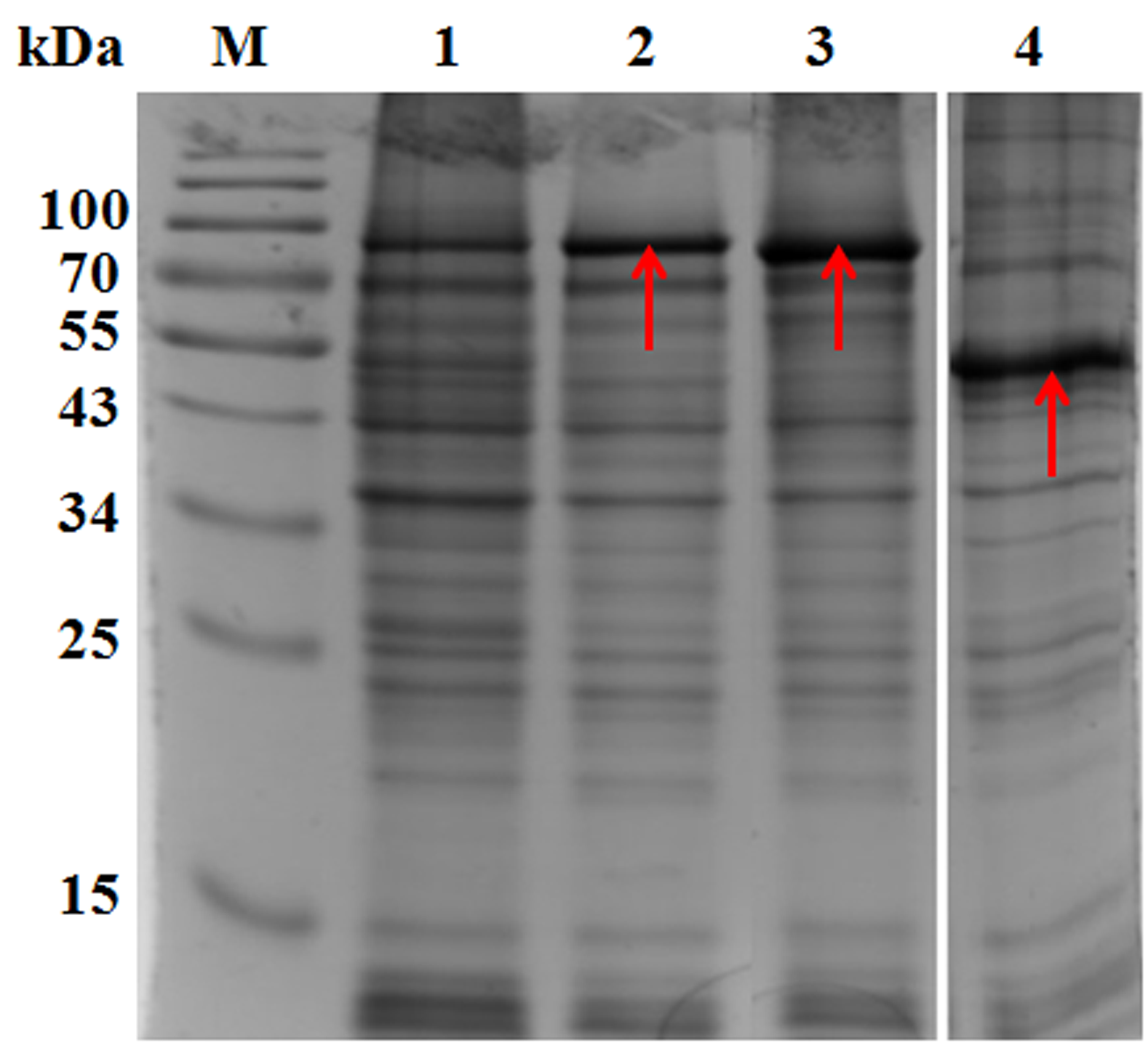

Supplement: S1 Fig — Lane 1: E. coli BL21 (DE3) transformed with pET-28a (+) induced by IPTG; Lane 2: E. coli BL21 (DE3) containing recombinant plasmid pIGN-PDHA induced by IPTG; Lane 3: E. coli BL21 (DE3) transformed with the pIGN-PDHB induced by IPTG. Lane 4: E. coli BL21 (DE3) transformed with pIGN induced by IPTG. Red marks, expressed recombinant fusion protein. (TIF) [file pone.0208745.s001.tif]
